# Evaluation of the Nephroprotective Activity of Crude Extract Root of *Rumex Abyssinicus Jacq* in Swiss Albino Mice With Gentamicin-Induced Nephrotoxicity: In Vivo Study

**DOI:** 10.1155/jt/4093111

**Published:** 2025-11-14

**Authors:** Asrat Tadelle Ewunetie, Banchamlak Teferi, Melkamu Siferih, Yadelew Yimer, Tiget Ayelgn Mengstie, Tewodros Shibabaw Molla

**Affiliations:** ^1^Department of Biomedical Science, College of Health Sciences, Debre Birhan University, Debre Birhan, Ethiopia; ^2^Department of Clinical Pharmacy, School of Pharmacy, College of Medicine and Health Sciences, University of Gondar, Gondar, Ethiopia; ^3^Department of Obstetrics and Gynecology, School of Medicine, Debre Markos University, Debre Markos, Ethiopia; ^4^Department of Biochemistry, School of Medicine, College of Medicine and Health Sciences, University of Gondar, Gondar, Ethiopia

**Keywords:** gentamicin, nephroprotective, nephrotoxicity, *Rumex abyssinicus*

## Abstract

**Introduction:**

Acute kidney injury represents a spectrum of diseases and is one of the most common threats to global public health. Gentamicin is the most common nephrotoxic antibiotic drug. Consequently, there is an urgent need to identify effective and safe therapeutic options from medicinal plants. Therefore, the purpose of this in vivo study is to evaluate the nephroprotective effects of the root of *Rumex abyssinicus Jacq* in Swiss albino mice exposed to gentamicin toxicity.

**Methods:**

A total of 30 mice were divided into five groups of six mice each. It comprised a normal control group (Group I) that received distilled water orally, Group II (100 mg/kg gentamicin-induced) without any intervention, and Group III–V that were experimental groups induced with 100 mg/kg gentamicin and treated with extracts of *Rumex abyssinicus* at the dose of 100, 200, and 400 mg/kg daily for 14 days. Blood and kidney tissues were taken for biochemistry and histological analysis on the fifteenth day.

**Result:**

*Rumex abyssinicus* extract treatment provided a significant nephroprotective effect (*p* < 0.001) against gentamicin-induced toxicity, as indicated by increased body weight and reduced kidney weight. In mice given 200 and 400 mg/kg of extract, the creatinine, urea, and uric acid measurements were decreased significantly compared to Group II. Furthermore, a similar dose of extracts showed the prevention of kidney damage via reduced tubular necrosis, glomerular congestion, and mononuclear infiltration, compared to the negative control, whereas mice given at the dose of 100 mg/kg extract showed no difference compared with Group II.

**Conclusion:**

This study explained that the extracts of *Rumex abyssinicus* might act as a potent free radical scavenger and restore the toxic effects of gentamicin and a potential nephroprotective agent.

## 1. Introduction

Aminoglycoside (AG) antibiotics, including gentamicin, amikacin, and tobramycin, are bactericidal antibiotics that inhibit the 30S subunit of both Gram-positive and Gram-negative bacterial ribosomes [1, 2] and have been introduced for clinical use in humans [3]. AGs are commonly used in the medical management of ocular, pulmonary, and intestinal infections [4] and are sometimes reserved for the management of complicated and multidrug-resistant infections. In particular, gentamicin is frequently and empirically prescribed in the management of neonatal sepsis (common etiologies are *Streptococcus* (43%) and *Escherichia coli* (29%) [Group B]) [5]. However, their use requires careful monitoring due to potential nephrotoxicity and ototoxicity, especially in vulnerable populations, such as neonates [6]. Given this, the kidney is the most vulnerable organ to toxic compounds because it receives approximately 20% of cardiac output, i.e., a significant volume of blood, and absorbs and concentrates hazardous toxin compounds within the renal tissues [7, 8].

Acute kidney injury (AKI), formerly called acute renal failure, is a clinical syndrome explained as a sudden and usually reversible alteration in kidney function or structure and usually a reduction in the glomerular filtration rate (GFR) [9] and typically lasts no longer than a week. This result is currently defined as an absolute increase in serum creatinine level of 0.3 mg/dL within 48 h or an increase of ≥ 1.5 times occurring within the previous 7 days [10–12]. Moreover, it also results in increments of blood urea nitrogen and other nitrogenous metabolic waste products that are normally excreted via the renal system. In addition, while creatinine is the most widely used serum biochemistry to assess for AKI, there are novel biomarkers for the detection of subclinical kidney injury prior to increase in creatinine level and include plasma or urine neutrophil gelatinase–associated lipocalin (NGAL, 25 kDa protein), serum kidney injury binding protein-1 (sKIM-1,104 kDa), serum cystatin C (sCysC) [13–15], and interleukin-18 before the increase in creatinine [16, 17]. Drugs that cause nephrotoxicity include several cancer therapies or chemotherapeutic agents, AG antibiotics, radiocontrast agents, nonsteroidal anti-inflammatory drugs, and immunosuppressants [18–20]. The rapid deterioration of kidney function is due to the toxic effects of such nephrotoxic substances [21].

Drug-induced AKI can occur through various pathogenesis mechanisms, notably proximal tubule complications arising from nephrotoxic or cytotoxic agents and their metabolites. Additionally, tubular obstruction is caused by crystals or casts formed from drugs. Furthermore, some drugs can trigger interstitial nephritis, an immune-mediated reaction that is classically independent of their dosage [22]. In sum, its clinical presentation ranges from a decrease in glomerular filtration rate (GFR) to nephritic syndrome and hydroelectrolytic disorders (HEDs) associated with glomerular and tubular damage [23, 24]. Therefore, AKI is a progressive decline of kidney function, which can lead to irreversible renal failure if not treated appropriately [25]. Nephrotoxicants have a variety of effects on the kidney, including blockage, intrarenal factors, and vasoconstriction, which lower the GFR [26].

Among many more drugs, gentamicin is one of the most widely used parenteral AG analogs with a broad-spectrum concentration-dependent bactericidal effect. However, its clinical application is significantly constrained by well-documented off-target tissue damage, particularly nephrotoxicity and ototoxicity [27–29]. Around 30% of patients treated with gentamicin, for more than 7 days, can experience renal injury of varying degrees [30]. Approximately 3%–5% of the introduced gentamicin is actively absorbed by the cells of the proximal tubule, causing necrosis of the S1–S2 segments of the proximal tubule [8, 31]. Moreover, gentamicin reduces the number and size of glomerular endothelial cell pores, also called fenestrae, resulting in decreased filtration. During transgenic nephrotoxicity, elevated calcium levels activate phospholipases, nucleases, and proteases that disrupt cell membrane function and cause further damage [32, 33]. Thus, the buildup of gentamicin in proximal convoluted tubules causes the activation of caspases, mitochondrial damage, tubular necrosis, and apoptosis production of harmful amounts of reactive oxygen species (ROS) [34, 35].

Medicinal plants are known nephroprotective agents that contain a wide variety of complex chemicals that give them healing properties [36]. According to estimates from WHO, approximately 80% of people get their basic medical care from traditional medicine, with a significant majority of those treatments being plant extracts [37].

It has been demonstrated that medicinal plants with nephroprotective effects can decrease the toxicity of medications when taken. Plants have renoprotective effects with various complex chemicals [38]. Plant secondary metabolite compounds have profound effects on renal physiology, including renoprotective effects [39].

*Rumex abyssinicus* (RA) Jacq (*R. abyssinicus*, known in Amharic as *Mekimeko*) belongs to the family Polygonaceae and is predominantly found across tropical Africa, mainly in Central and Eastern Africa [12, 40], with approximately 200 species [41]. RA is an important place in the history of Ethiopian traditional medicine, being used in the treatment of numerous human diseases.

### 1.1. Phytochemical Studies of RA

Most of the species of Polygonaceae contain a large number of bioactive compounds, such as anthraquinones, flavonoids, terpenes, naphthalenes, stilbenoids, steroids, flavonoid glycosides, leucoanthocyanidins, and phenolic acids [42]. The phytochemical studies of RA extract revealed that the presence of tannins, flavonoids, phenols, quinones, alkaloids, carotenoids, phlorotannins, terpenoids, naphthalene, stilbenoids, steroids, glycosides, saponins, fats, and oils was reported in several scholars [37, 43, 44].

### 1.2. Medicinal Uses of RA

Traditionally, different parts of *Rumex* species have been used to treat several health problems, such as infections, diarrhea, constipation, mild diabetes, edema, jaundice, skin, liver, and gallbladder disorders and inflammation, and they are also used as antihypertensive, diuretic, kidney disorder, and analgesic preparation [45–47]. It is also used to treat malaria, gonorrhea, poisoning, hepatitis, constipation, sciatic neuralgia, hypertension, migraine, rheumatism, breast cancer, stomach distention, earache, hepatic diseases, hemorrhoids, typhus, rabies, and wounds [48]. RA was reported to demonstrate a wide range of bioactivities, including antibacterial, antiviral [49], cholinesterase inhibitor [50], anthelmintic [51], wound healing, anti-inflammatory [49], antioxidant [52], lytic activities against zoospores, Trypanosomacidal, and antitumor [53]. In addition, the RA showed a chemopreventive potential against dimethyl hydrazine–induced colon carcinogenesis in rats, and it also displayed anticancer activity in prostate, brain, breast, and leukemia cell cultures [54].

The Polygonaceae family medicinal plant is used to treat renal detoxification and kidney stones [55, 56]. Traditionally, RA Jacq is used for the treatment of urological disorders (kidney problems, kidney infections, etc.) in Ethiopia [57]. The root part of RA Jacq used to treat kidney problems in different parts of Ethiopia, including the study area [58].

Furthermore, it is used for diuretic and kidney infections in Ethiopia [47]. The root and the stems are used to treat diabetes and hepatitis [59], hypertension, wounds, cancer, tuberculosis, tooth infections, abdominal problems, and stomach pain [12, 60, 61].

Even though several studies have been conducted on different parts of RA, no scientific study has demonstrated and reported the nephroprotective effects of its root extract. Therefore, this study was conducted to bridge the aforementioned gaps through the examination of antioxidant activity and nephroprotective effect of the root extract of RA in gentamicin-induced nephrotoxic Swiss Albino mice.

## 2. Materials and Method

### 2.1. Chemicals, Reagents, and Instruments

To carry out this study, various drugs, reagents, and instruments were utilized. The chemicals and reagents included methanol, distilled water, gentamicin, formalin, paraffin wax, diethyl ether, DPPH, FeCl_3_ (BDH chemical pool), gallic acid, ascorbic acid (Blulux Laboratories, India), and quercetin. Equipment or materials used included a mechanical grinder, filter paper, gauze, plastic sample holder, rotary evaporator (Yomato, USA), heating bath, volumetric flasks, funnel, mortar and pestle, separatory funnel, spoons, beakers, measuring cylinders, and spatulas. Additionally, serum separator tubes (SSTs), vacuum blood collection tube gel and clot activators, a centrifuge machine (West Sussex, UK), a fully automated serum analyzer, oral gavages, frost-ended slides, syringes, desiccators, a heater, refrigerator, surgical and disposable gloves, scissors, forceps, tissue cassettes, a tissue processor, a cool plate, a water bath, labelers, markers, microscope, and mice cages were used.

### 2.2. Collection and Extraction of RA Plant

**Figure 1:**
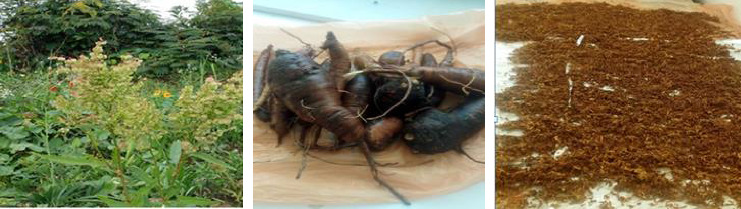
Photograph of different parts of *R. abyssinicus Jacq* plant (leaf, seeds) collected from the sample area and chopped root in the laboratory session (steam, root, and seed from right to left).

The root part of RA shown in Figure 1 was collected from Sekele Mariam Forest, Dembecha Woreda of the West Gojam Zone, Amhara region, Ethiopia, in May 2023, to the northwestern 350 km of Addis Ababa, the capital city of Ethiopia [62]. The plant was identified and authenticated by a botanist with a “voucher number: AT-001” and has been given and deposited in the herbarium of Biology Department, College of Natural and Computational Science, Debre Markos University, Ethiopia.

The freshly harvested roots were thoroughly cleaned with deionized water and air-dried at room temperature and subsequently chopped by using a laboratory knife. The dried chopped root will be powdered using a mechanical grinder and then passed through a 0.1-mm sieve and stored in an air-tight container. The amount of 600 g of dried powder root was extracted by using three successive maceration extraction techniques with 80% methanol solvent. The powder was soaked for 3 days by using 1:5 ratio of a gram of sample and ml of 80% methanol solvent, accompanied by occasional shaking and stirring [63, 64]. Filtration was conducted via Whatman filter paper No. 1 and No. 42 and then evaporated by a rotary evaporator, and residual solvent was removed at low temperature under reduced pressure.

The concentrated extract obtained through the process is termed as a crude extract. The resulting dried powdered mass was placed into a glass vial, appropriately labeled, and stored in a refrigerator at 4°C for further experimental use.

The extraction yield percentage was determined using the following formula:

(1) $$\begin{array}{c} \text{percentage yield}=\left(\frac{\text{Wt}.\text{ of dried and collected extract }}{\text{Wt}.\text{ of the sample }\left(\text{powder}\right)}\right)\times 100 \end{array}$$

### 2.3. Phytochemical Analysis

Preliminary phytochemical screening was conducted to evaluate the presence of key bioactive constituents in the crude extract of RA leaves. This qualitative assessment was performed via standard procedures and identified compounds such as saponins, flavonoids, alkaloids, tannins, phenols, steroids, quinones, anthraquinones, and glycosides that might be responsible for the biological activity [43, 65–67].

### 2.4. In Vitro Antioxidant Study

#### 2.4.1. DPPH (2,2-Diphenyl-1-Picrylhydrazyl) Free Radical Scavenging Assay

To assess antioxidant activity, a series of extract concentrations (25, 50, 100, 200, and 400 mg/L) was pipetted out from 1000 ppm stock solution. Each concentration was mixed with 100 μL of 0.2 mM methanolic DPPH solutions. The absorbance reading was taken at 517 nm using a UV spectrophotometer (Model Cary-60 Agilent Technologies). For comparison, ascorbic acid was used as the reference (positive control) and prepared at the same concentrations as the extract. The percentage inhibition of DPPH by the extract was then calculated using the following equation [68]:

(2) $$\begin{array}{c} \text{DPPH scavenging effect }\left(\%\text{ inhibition}\right)=\left(\frac{A\text{ pure DPPH}-A\text{ sampl}e}{A\text{ pure DPPH}}\;\right)\times 100. \end{array}$$

#### 2.4.2. Ferric Reducing Antioxidant Power (FRAP) Assay

The reducing power of the crude extract was evaluated using the standard procedure. Equal volumes (3 mL each) of the root extract at different concentrations (25, 50, 100, 200, and 400 mg/L), phosphate buffer solution (PH = 6.6, 0.2 M), and 1% potassium hexacyanoferrate (K_3_Fe (CN)_6_) were mixed together. The solution was incubated at 50°C in the water bath for 20 min. Then, 3 mL of 10% trichloroacetic acid was added to the mixture to terminate the reaction and centrifuged at 1,000 rpm for 10 min. The upper layer was mixed with 1 mL of 0.1% FeCl_3_ and 5 mL of distilled water solution. The reaction mixture was incubated at room temperature, and the absorbance was subsequently measured at 700 nm, with a Blank solution as a reference:

(3) $$\begin{array}{c} \text{percentage}\left(\%\right)\text{reduction power}=\left(\frac{A\text{ sample}-A\text{ blank}}{A\text{ sample}}\right)\times 100, \end{array}$$

where A sample = sample absorbance and A blank = blank absorbance.

### 2.5. Pre-Experimental Animal Care

A total of 35 healthy Swiss albino mice of both sexes, 8–10 weeks and (30–40 g), were used in the study. Five female mice were used for the acute toxicity test, and 30 male mice were used for the nephroprotective experiments.

Male mice are more susceptible to drug-induced nephrotoxicity such as gentamicin. The cyclic hormonal variations in female mice can impact biochemical levels and potentially offer some protective effects. Hormones like testosterone might be involved in regulating the vulnerability of male mice to nephrotoxicity [12].

Experimental mice were obtained from the animal house of the Pharmacology Department, College of Medicine and Health Science, University of Gondar, for the study. The mice were housed under standard environmental conditions at room temperature (22°C ± 2°C) with a 12 h light/12 h dark cycle in ventilated plastic cages. There was free access to pellet diet and water ad libitum until the date of laboratory work [69].

Six mice were housed in a single polypropylene transparent cage. Cages were labeled, and each mouse was given a specific code on the tail. All experimental procedures were conducted according to internationally recognized ethical guidelines and were approved by the Institutional Ethics Committee [70]. Prior to the start of the experiment, the animals were allowed to acclimatize to the laboratory environment for a period of 1 week.

## 3. Experimental Design

### 3.1. Acute Toxicity Testing/Limit Dose Test

A limit test or acute toxicity study was conducted according to the Organization for Economic Co-operation and Development (OECD No. 425) guideline to evaluate the potential toxicity of the extracts as well as to select safe doses for the evaluation of nephroprotective activity [71]. A previous study conducted acute toxicity in crude methanol rhizome extract of RA was administered orally at a single dose of 2,000 mg/kg body weight [72]. Five female mice were kept fasting overnight before experimentation, and the body weight of the mice was noted. One female mouse was administered first at a limited test dose of 2000 mg/kg extract and then dosed four additional animals sequentially [73]. After administration, mice were observed continuously for gross behavioral changes for 24 h and 72 h in regular intervals for 14 days.

### 3.2. Selection of Gentamicin and Extract Dose

Gentamicin was injected intraperitoneal (i.p.) in the animals at a dose of 100 mg/kg (body weight), which is the normal dose to induce nephrotoxicity [74]. The crude extract will be suspended in normal saline (0.9% NaCl), and each group of animals was given different doses of extract administered orally using plastic tube [72]. The dosage of the extract was determined according to the acute toxicity study following the OECD guideline that states 2 mL/100 g of body weight of the animal. The first dose was half of the second dose (100 mg/kg), the second dose was one-tenth of the limited dose (200 mg/kg), and the third dose was twice the second dose (400 mg/kg) [75].

### 3.3. Experimental Animals' Grouping and Treatment Dose

The animals were randomly divided and assigned into five experimental groups. Each plastic cage contains six mice. The experimental protocol was described by Chinnappan et al. with minor modifications [76] to align with this study, as shown in Table 1.

### 3.4. Biochemical Analysis

The body weight of the mice was measured on the 0^th^ day and 14^th^ day of the treatment by using an electronic balance. Following an overnight fast after the final dose, the animals were sacrificed. The relative kidney weight was calculated for each mouse as a percentage of the kidney weight to the body weight of the mouse. Anesthesia was induced using diethyl ether, and approximately 2-3 mL of blood samples was withdrawn by cardiac puncture and transferred to an SST. The samples were centrifuged at 3000 rpm for 10 min. Serum levels of creatinine, blood urea, and uric acid were then measured using a clinical chemistry autoanalyzer (Jor Lab bio-2000) following the manufacturer's protocol [12].

### 3.5. Histopathological Examination

In experimental mice sacrificed after being anesthetized with diethyl ether, blood samples were withdrawn by cardiac puncture [77]. Each mouse was placed in a supine position on a dissection board, and the limbs were secured. The kidney was surgically removed, cleaned of the surrounding connective tissues, and rinsed with normal saline to remove any blood residues. The pieces of kidney from each group were washed with normal saline, fixed immediately in 10% neutral buffered formalin for 24 h, dehydrated in graded (50%–100%) alcohol, embedded in paraffin, and sectioned into 5-μm-thick slices using a rotary microtome. These sections were stained with hematoxylin and eosin for histologic examination. Subsequently, the stained slides were coded and assessed by a blinded pathologist to the treatment groups, via a light microscope for the assessment of histopathological changes at the University of Gondar Specialized Hospital.

### 3.6. Statistical Analysis

Data were entered into SPSS 25.0 for analysis. Statistical data analysis was done using one-way ANOVA with a significant level set at *p* < 0.05. Where a significant difference was detected between groups, Tukey's post hoc test was applied to identify specific group differences. Results were expressed as mean ± standard deviation (SD) and presented in the form of tables and figures.

## 4. Results

### 4.1. Percentage Yield of Extracts

The extraction of dried RA root using 80% methanol solution was carried out for three consecutive rounds of cold maceration. From 600 g of air-dried, powdered root material, a crude extract yield of 76.60 g was obtained:

(4) $$\begin{array}{c} \text{percentage yield}=\left(\frac{76.60\;g}{600\;g}\right)\times 100=12.8\%. \end{array}$$

The percentage yield of crude RA root extract was calculated to be 12.8% (weight by weight).

### 4.2. Antioxidant Activity (DPPH) Assay

**Figure 2:**
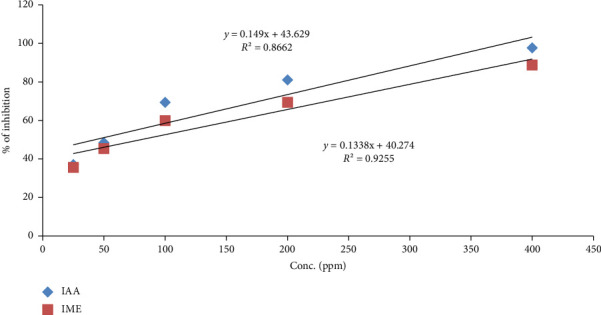
The calibration curve of IC_50_ value of ascorbic acid and methanol extract of *Rumex abyssinicus* root in the DPPH assay. IAA: inhibition (IC_50_) with ascorbic acid, IME: inhibition (IC_50_) with methanol extract of *Rumex abyssinicus* root.

The IC_50_ value of the extract of RA root and ascorbic acid for DPPH radical scavenging activity was found at 72.69 and 42.78 mg/L, respectively. In a dose-dependent manner, free radical scavenging activity increased. Thus, Figure 2 below indicates that there is a moderate antioxidant potential of the plant extract.

### 4.3. FRAP Assay

**Figure 3:**
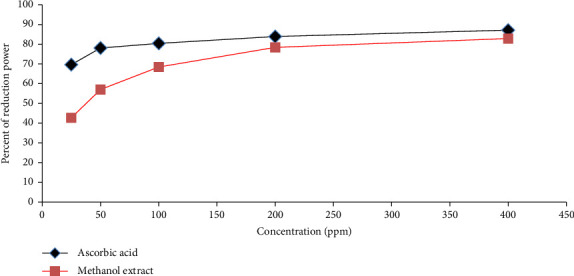
Demonstrating dose-dependent ferric reducing antioxidant power (FRAP) of *Rumex abyssinicus* root extract compared to ascorbic acid. Black line: *Rumex abyssinicus* extract, red line: ascorbic acid (standard).

The FRAP assay revealed a dose–response relationship, with the absorbance increasing as the concentration increased for RA root extract. The antioxidant compounds of the extract donate an electron that is responsible for neutralizing reactive ferric (Fe^3+^) to ferrous (Fe^2+^) transformation. FeCl_3_ was added to the ferrous form, resulting in the creation of a blue-colored complex.  Figure 3 illustrated a dose-dependent enhancement in antioxidant activity, where higher concentrations of the RA extract result in greater absorbance, indicating stronger reducing potential. The absorbance and percentage of the reduction potential of the extract increased with the dose, confirming its strong reducing ability. Thus, in terms of contributing to its nephroprotective effects, the RA extract was comparable, though slightly lower than the standard antioxidant (ascorbic acid).

### 4.4. Effect of RA on General Parameters in Experimental Animals

#### 4.4.1. Effects on Body and Kidney Weight Change

At the end of the experiment, body weight change and kidney weight were determined for each group of animals. The impact of RA on general parameters of gentamicin-induced nephrotoxicity in both healthy control and experimental mice was evaluated in each group and summarized in Table 2. Body weight measurements were recorded on the 0^th^ day (prior to treatment) and the 14^th^ day (after the experiment period). After gentamicin treatment (Group II), the mice demonstrated significant (*p* < 0.001) body weight loss and increased kidney weight, indicating kidney damage (inflammation and swelling) compared to the healthy mice. Conversely, mice treated with 200 and 400 mg/kg of the RA extract (Group IV (*p* < 0.01) and Group V (*p* < 0.001)), respectively, demonstrated a significant improvement in body weight and reduction in kidney weight compared to the gentamicin-exposed Group II, suggesting potential protective effects of the extract. However, Group III-treated experimental animals have no significant differences from Group II GM-induced group. These results indicate a dose-dependent nephroprotective particularly at 200 and 400 mg/kg effect of the extract, with better outcomes observed at higher doses.

### 4.5. Biochemical Test

#### 4.5.1. Effect of RA Extracts on Serum Creatinine, Blood Urea, and Uric Acid Levels

The gentamicin-induced mice (Group II) exhibited significantly raised creatinine levels (*p* < 0.001) compared to the control mice. Treatment with methanol extracts of RA at the dose of 200 and 400 mg/kg (*p* < 0.001) recorded lower creatinine levels than gentamicin treatment. In contrast, the 100 mg/kg extract treatment group (Group III) also showed a significantly higher creatinine level compared with the control group (*p* < 0.01), Group IV, and Group V (*p* < 0.001) and did not differ from the group under gentamicin treatment, dictating minimal nephroprotection at low dose. The percentage reduction in serum creatinine level in 200 and 400 mg/kg groups compared to gentamicin was 11.8% and 23.53%, respectively. This shows RA extract has a dose-dependent effect, as the dose of extract increases, a greater reduction of serum creatinine level. Similarly, blood urea levels were recorded significantly higher in gentamicin-induced Group II and Group III compared to the control group (*p* < 0.001). However, treatment with RA extract at doses of 200 and 400 mg/kg showed lowered blood urea levels compared to Group II (*p* < 0.001) and Group III (*p* < 0.05), as shown in Table 3.

However, there was no significant difference in uric acid concentration among the plant extracts treated groups. The 100 mg/kg extract-treated group (*p* < 0.01) and gentamicin-induced group (*p* < 0.001) showed increased uric acid levels compared to the normal control. The 200 and 400 mg/kg treated groups showed significant differences (*p* < 0.001) with GM-induced Group II mice.

### 4.6. Histopathology Studies

**Figure 4:**
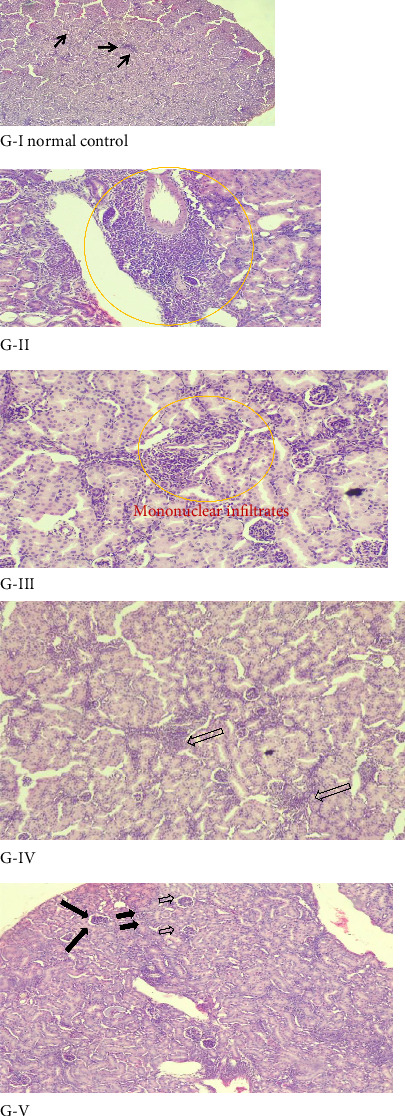
Histopathological examination of kidney section from normal control, gentamicin-treated, and dose-dependent *R. abyssinicus*–treated mice (10 and 20x, stained with hematoxylin and eosin).

The examination of the kidney sample of mice under an electronic microscope reveals observable differences in kidney morphology among the normal control (Group I), the GM-induced kidney disease mice (Group II), and the extract treatment groups.  Figure 4 shows that the kidney samples in Group I (control group) show normal glomeruli with intact Bowman's capsule, normal proximal and distal convoluted, and no capillary congestion. Mice given GM (100 mg/kg) intraperitoneally for 8 days developed acute renal injury as seen by significant tubular necrosis and inflammation signs, such as glomerular congestion, mononuclear infiltration, and blood vessel congestion (Table 4 and Figure 4). However, in the RA extract-treated group of mice, a dose-dependent variation in regenerative capability was observed between Group IV and Group V treated with 200 and 400 mg/kg, respectively. This showed a remarkable reduction in tubular necrosis and inflammation signs such as glomerular congestion, mononuclear infiltration, and blood vessel congestion compared to the GM-treated group. The nephroprotective effect is better with 400 mg/kg extract than with 200 mg/kg. The 400 mg/kg treated mice showed either no histologic abnormalities at all or very few. Together, the histopathologic examination indicates a dose-dependent nephroprotective effect of the RA extract against gentamicin-induced kidney toxicity.

GI showed a photomicrograph of a normal control, where normal glomerulus, normal Bowman's space, and normal renal tubules. GII showed a photomicrograph of a negative control (mice received 100 mg/kg gentamicin only), where pathological damage shows in the mononuclear cell, plasma cell, Bowman's space dilatation, glomerular congestion, severe inflammatory cells, and tubular necrosis (circled). G III showed a photomicrograph of mice received 100 mg/kg RA extract+100 mg/kg gentamicin, where pathological damage was shown by circle moderate mononuclear infiltration, moderate glomerular congestion, mild inflammatory cells, and tubular necrosis (circled). GIV showed a photomicrograph of mice received 200 mg/kg RA extract + 100 mg/kg gentamicin, where pathological damage was shown by an arrow, mild glomerular congestion and inflammatory cells. GV showed a photomicrograph of mice received 400 mg/kg RA extract + 100 mg/kg gentamicin, where pathological damage shown by arrow a mild glomerular congestion. However, other histological features in Group V are almost comparable with those of the normal group.

### 4.7. Limitations of the Study

The current study utilized only the crude extract of RA, which prevented the identification of specific active compounds and the detailed mechanisms underlying its nephroprotective effects. Additionally, limited biochemical parameters were measured. Due to financial limitations, the scope of our research was hindered, and we faced difficulties in conducting laboratory experiments involving treatment with silymarin. Therefore, further investigations are necessary to isolate and identify the active ingredients and to clarify the mechanisms responsible for the plant's protective effects. In this study, there were no analyzed novel biomarkers and antioxidant enzymes analyzed in renal tissues or in serum. Further investigations require novel biomarker renal functional tests, such as kidney injury biomarker (KIM1, NGAL, IL-18) and kidney tissue antioxidant enzymes.

## 5. Discussion

Gentamicin is the most widely prescribed and used parenteral AG analogs, having a concentration-dependent bactericidal effect [78]. It prevents the formation of proteins in the bacterium. However, these perceived advantages are frequently overshadowed by the well-known adverse effects of nephrotoxicity [79]. Gentamicin-induced nephrotoxicity involves a complex and sophisticated process, triggering numerous cellular responses via involving multiple mechanisms that culminate in renal damage and tissue necrosis [80]. Reactive oxidative stress is a major cause of GM-induced AKI. Herbal remedies have potent protective properties against AKI caused by pharmaceutical agents [81], e.g., gentamicin.

The present study revealed that the methanolic extract of RA root possesses potential nephroprotective properties, effectively counteracting gentamicin-induced kidney injury in mice. The extract contains a high concentration of a significant phytochemical profile and flavonoids, which are known for their potent antioxidant properties via free radical scavenging and capable of neutralizing reactive free radicals and mitigating oxidative stress.

Thus, a common mechanism of action of flavonoids is to reduce inflammation and oxidative stress in the kidney [82] and reported to reduce gentamicin-induced renal injury [38]. Current information regarding the effect of flavonoids on renal function and the associated mechanisms of action indicates that flavonoids have a significant impact on renal physiology, have diuretic and natriuretic effects, and may also be associated with AKI and oxidative stress factors in the kidney. It has been suggested that it has nephroprotective effects against toxicity [83].

In this study, the antioxidant capacity of the extract of RA root was assessed by two different assays: first using DPPH scavenging activity and second via FRAP. The two methods were used to evaluate the antioxidant capacity of the extract of RA. Both DPPH and FRAP radical scavenging assay collectively demonstrated that a significant antioxidant activity in the 80% methanol extract of RA. This activity exhibited a dose-dependent relationship, and antioxidant activities were enhanced with increasing the concentration of the extracts consistent with previously reported [52] research. Phenolic and flavonoid compounds are important constituents of a plant, whose hydroxyl groups confer free radical scavenging potential [84, 85]. The extract of RA in our study demonstrated significant free radical scavenging activity with an IC_50_ value of 72.69 mg/L in the DPPH assay. This has a significant difference from the aqueous extract *R. sativus* (30.04 mg/L), lower IC_50_ reported by Zrouri et al. and higher than *T. microphylla* (111 ± 0.02 mg/L) reported by Bencheikh et al.

The extract demonstrates a significant ferric reducing ability and a strong counteracting antioxidative activity. The reducing properties associated with the presence of compounds work by donating a hydrogen atom to break the free radical chain. The ability of extracts to reduce iron (FRAP) suggests that they contain compounds that serve as electron donors, converting free radicals to more stable products and ending radical chain reactions [86]. In RA root extract, the FRAP assay revealed a favorable connection between reducing power and phenolic content [87]. Together, a dose-dependent ferric reducing ability in FRAP assay supports the therapeutic potential of the extract. Table 2 maps the impact of extract on weight gain and relative kidney weight in GM-induced mice. Daily intraperitoneal administration of the mice by the GM (100 mg/kg) produced a significant reduction of body weight gain and an increase (*p* < 0.001) in relative kidney weight, compared to normal control mice. Extracts of RA treatment at doses of 200 and 400 mg/kg show a significant difference compared with GM-induced mice, whereas the 100 mg/kg dose did not achieve statistical significance. This dose-dependent nephroprotective efficiency is consistent with the findings reported by Bencheikh et al. and Abebe et al. [88, 89]. Batileta et al. explained that a possible mechanism for weight loss may be increased production of proinflammatory cytokines [90].

The significant difference between the relative weights of kidneys exposed to GM alone (100 mg/kg) and other groups indicates that gentamicin accumulates in the kidneys, causing renal inflammation, inflammatory cell infiltration, interstitial edema, and decreased food intake, thereby causing body weight loss. Because kidney damage leads to weight loss, the accumulation of GM in kidney tissue results in the loss of tubular cells responsible for water reabsorption, leading to dehydration and subsequent weight loss [91]. After the course of treatment with GM-induced mice, we demonstrate with similar mechanism that leads to an increase in the relative weight index of the kidney. However, treatment groups of mice with RA extracts result in a dose-dependent reduction in kidney weight relative to body weight. This depicts that the extract is attributed to the anti-inflammatory effect that negotiates inflammation and reduces edema. Therefore, the effect of our extract in decreasing the relative weight of kidneys may suggest its protective role in limiting the progression of GM-induced tissue damage [76].

In the current study, mouse nephrotoxicity was induced via administration of gentamicin daily. Then, after treatment, a marked elevation of serum creatinine, BUN, and serum uric acid levels (*p* < 0.001) was observed in the negative control Group II mice compared to the group that received 20 mL/kg of distilled water. Urea and uric acid are products of amino acid metabolism and nucleic acids, respectively, which serve as key indicators for renal excretory function [69, 92, 93].

The current investigation explores the protective role of RA against kidney injury in an established gentamicin-induced nephrotoxicity mouse model. Several studies have reported that AGs (gentamicin) are classic antibiotics that can cause nephrotoxicity through the induction of ROS [94]. This injury is typically explained by increased blood levels of creatinine, urea, and uric acid, along with marked damage to the proximal tubule and necrotic cell deaths followed by tissue swelling and kidney dysfunction [94].

The reduction of serum biochemistry levels observed in the groups of mice receiving 200 and 400 mg/kg of RA extract, compared to the gentamicin-induced group, suggests a protective effect of the plant extract. These polyphenols and flavonoids are known to mitigate gentamicin-induced nephrotoxicity by enhancing the activity of antioxidant enzymes, including superoxide dismutase (SOD), catalase (CAT), and glutathione peroxidase (GPx) activity. These findings are in agreement with the earlier studies [69]. There is no significant difference between low-dose extract 100 mg/kg with GM-induced group. Furthermore, RA extract at the highest dose (400 mg/kg) demonstrated a significant reduction in serum creatinine (0.53 ± 0.07 mg/dL, *p* < 0.01), blood urea nitrogen (63.47 ± 0.54 mg/dL), and uric acid (3.22 ± 0.53 mg/dL) levels (*p* < 0.001) compared with the GM-induced control (Group II mice). Similar results were reported by Hasan et al. who demonstrated nephroprotective effects in gentamicin-induced nephrotoxic albino mice. Likewise, an aqueous extract for M. peregrine 500 and 1000 mg/kg dose-treated mice group recorded a decrease in serum creatinine, blood urea, and uric acid levels [68]. This outcome is also supported by the studies conducted by Dubiwak et al. on nephrotoxicity-induced Swiss albino mice [88, 89]. Together, serum Cr, BUN, and uric acid levels reducing the effects of methanol extract of RA might be due to its antioxidant, anti-inflammatory, and free radical scavenging effect.

The histopathological observation in this study was consistent by the biochemical and antioxidant findings. In this experiment, mice treated (Group II) with gentamicin exhibited extensive renal tubular necrosis, glomerular congestion, and widespread infiltration of inflammatory cells, in line with several reports [76, 95]. The observed renal injury may be due to gentamicin-induced upregulation of proinflammatory cytokines and the recruitment of macrophages and neutrophils into renal tissue, leading to exuberant inflammatory cascade [85], and it reflects AKI mediated with drug-induced oxidative stress and overwhelmed inflammation. However, the extensive histopathological alterations were reduced in mice treated with RA *extracts*. Mice administered 200 and 400 mg/kg extract of RA had a beneficial effect on the renal system compared to treatment with GM-induced. Moreover, the Group V mice (400 mg/kg) demonstrated a dose-dependent effect, with histological features closely resembling those of the normal control mice. Overall, these histological findings strongly support the biochemical results and confirm the nephroprotective efficacy of RA extract in a dose-responsive manner.

## 6. Conclusion

Taken together, based on our study, the methanolic extract of the root of RA has an appreciable source and is rich in promising antioxidant phytochemical constituents, such as phenolic compounds, flavonoids, and other secondary metabolites, which are efficient antioxidant capacity in DPPH and FRAP assays. The nephroprotective effects on mice treated with RA *extract* possess potential protective activity against gentamicin-induced kidney toxicity in Swiss Albino mice in a dose-dependent manner. These protective effects were evidenced by improvements in renal function tests, restoration of normal histologic architecture of renal tissue, and a reduction of relative kidney weight.

Overall, this study provides scientific support for the traditional use of RA, a medicinal plant, and opens new avenues for molecular-level research focused on the isolation and purification of various known and unknown bioactive constituents.

## Figures and Tables

**Table 1:** Grouping of experimental animals and treatment protocol (14 days of study period).

Group of mice	Category	Dose of treatment administration
I	Normal control	20 mL/kg distilled water p.o. for 14 days
II	GM-induced (negative control)	20 mL/kg distilled water p.o. + 100 mg/kg GM i.p. daily for the last 8 days
III	RA100	100 mg/kg b.wt of RA extract p.o. daily and 100 mg/kg GM i.p. after 1 h the RA treatment for the last 8 days
IV	RA200	200 mg/kg b.wt of RA extract p.o. daily and 100 mg/kg GM i.p. after 1 h the RA treatment for the last 8 days
V	RA400	400 mg/kg b.wt of RA extract p.o. daily and 100 mg/kg GM i.p. after 1 h the RA treatment for the last 8 days

**Table 2:** Effects of *R. abyssinicus* extracts on body weight change and absolute and relative kidney weights.

Groups treatment	Body weight (g.)		Change in body wt. (g.)	Kidney wt. (g.)	Relative kidney wt. (%)
	0^th^ day	14^th^ day			
Control	31.43 ± 3.32	36.97 ± 2.04	5.53 ± 0.29	0.433 ± 0.05	1.17 ± 0.001
Gentamicin	31.00 ± 1.88	32.02 ± 1.69	1.02 ± 0.64^∗∗∗a^	0.65 ± 0.1^∗∗∗a^	2.03 ± 0.003^∗∗∗a^
RA extract 100 mg/kg	32.62 ± 5.05	35.40 ± 4.01	2.78 ± 0.45^∗∗a,ns^	0.500 ± 0.09^∗b^	1.4 ± 0.0015^∗b^
RA extract 200 mg/kg	30.03 ± 3.75	33.57 ± 2.93	3.53 ± 0.34^∗b^	0.483 ± 0.08^∗∗b^	1.44 ± 0.0021^∗∗b^
RA extract 400 mg/kg	30.23 ± 2.31	34.63 ± 1.75	4.67 ± 0.34^∗∗b^	0.450 ± 0.05^∗∗∗b^	1.31 ± 0.002^∗∗∗b^

*Note:* Values are expressed in mean ± standard error of the mean (*n* = 6). One-way analysis of variance (ANOVA) and the Tukey multiple comparison test were used to examine the data.

^a^Significant compared with the control group (Group I).

^b^Significant compared with GM-induced group (Group II).

^ns^Not significant compared with GM-induced group (Group II).

^∗^ *p* < 0.05.

^∗∗^ *p* < 0.01.

^∗∗∗^ *p* < 0.001.

**Table 3:** Effect of extracts of *R. abyssinicus* on serum creatinine, blood urea, and uric acid levels.

Treatment	Serum creatinine (mg/dL)	Blood urea (mg/dL)	Uric acid (mg/dL)
Control GI	0.51 ± 0.04	53 ± 0.15	2.80 ± 0.44
Gentamicin-induced GII	0.65 ± 0.05^a∗∗∗^	95 ± 0.13^∗∗∗a^	5.68 ± 0.40^∗∗∗a^
RA100 mg/kg extract GIII	0.63 ± 0.06^∗∗a,ns^	84 ± 0.34^∗∗∗a,ns^	3.74 ± 0.50^∗∗a,∗∗∗b^
RA200 mg/kg extract GIV	0.59 ± 0.05^∗∗bc^	65.83 ± 0.0.24^∗∗∗b,∗c^	3.40 ± 0.34^∗∗∗b^
RA400 mg/kg extract GV	0.53 ± 0.07^∗∗bc^	63.47 ± 0.54^∗∗∗b,∗c^	3.22 ± 0.53^∗∗∗b^

*Note:* Values are presented as mean ± standard error of the mean (SEM) for each group (*n* = 6). ^∗^*p* < 0.05, ^∗∗^*p* < 0.01, and ^∗∗∗^*p* < 0.001 indicate statistically significant. The results were analyzed by one-way analysis of variance (ANOVA) followed by the Tukey multiple comparison test.

^a^Significant compared with the control group (Group I).

^b^Significant compared with GM-induced group (Group II).

^c^Significant compared with Group III.

^ns^Not significant compared with GM-induced group (Group II).

**Table 4:** Histopathological features of the kidney tissues of mice with gentamicin-induced nephrotoxicity and different treatment groups with variable doses of *Rumex abyssinicus* root extract.

Histological feature	Group I	Group II	Group III	Group IV	Group V
Tubular necrosis	−	+++	+	−	−
Inflammatory cells	−	+++	+	+	−
Blood vessel congestion	−	++	+	−	−
Glomerular congestion	−	++	++	+	+
Interstitial edema	−	++	+	−	−
Bowman's space dilation	−	+	−	−	−
Mononuclear infiltration	−	++	++	−	−

*Note:* Kidney sections were scored as none (−), mild (+), moderate (++), and severe (+++).

## Data Availability

The data that support the findings of this study are available from the corresponding author upon reasonable request.

## Nomenclature

UOG: University of Gondar
BUN: Blood urea nitrogen
Gm: Gentamicin
SOM: School of medicine
AKI: Acute kidney injury
P.O: Oral administration
I.P: Intraperitoneal administration
RA: *Rumex abyssinicus*
G: Gram
Mg: Milligram
kg: Kilogram
Wt: Weight
